# Molecular identification and antigenic characterization of a merozoite surface antigen and a secreted antigen of *Babesia canis* (BcMSA1 and BcSA1)

**DOI:** 10.1186/s13071-016-1518-1

**Published:** 2016-05-03

**Authors:** Mo Zhou, Shinuo Cao, Yuzi Luo, Mingming Liu, Guanbo Wang, Paul Franck Adjou Moumouni, Charoonluk Jirapattharasate, Aiko Iguchi, Patrick Vudriko, Mohamad Alaa Terkawi, Mario Löwenstein, Angela Kern, Yoshifumi Nishikawa, Hiroshi Suzuki, Ikuo Igarashi, Xuenan Xuan

**Affiliations:** National Research Center for Protozoan Diseases, Obihiro University of Agriculture and Veterinary Medicine, Obihiro, Hokkaido 080-8555 Japan; Harbin Veterinary Research Institute, CAAS-Michigan State University Joint Laboratory of Innate Immunity, State Key Laboratory of Veterinary Biotechnology, Chinese Academy of Agricultural Sciences, Maduan Street 427, Nangang District, Harbin, 150001 PR China; Megacor Diagnostik GmbH, Hoerbranz, Vorarlberg A-6912 Austria

**Keywords:** *Babesia canis*, Canine babesiosis, BcMSA1, BcSA1, ELISA, Immunochromatographic tests

## Abstract

**Background:**

*Babesia canis* is an apicomplexan tick-transmitted hemoprotozoan responsible for causing canine babesiosis in Europe and west Asia. Despite its importance, there is no known rapid diagnostic kit detection of *B. canis* infection in dogs. The present study identified two novel antigens of *B. canis* and used the recombinant antigens to establish a rapid, specific and sensitive serodiagnostic technique for detection of *B. canis* infection.

**Methods:**

A complementary DNA (cDNA) expression library was constructed from the mRNA of *B. canis* and immunoscreened using *B. canis-*infected dog sera. The cDNAs encoding a merozoite surface antigen and a secreted antigen protein were identified and designated as BcMSA1 and BcSA1, respectively. The recombinant BcMSA1 and BcSA1 (rBcMSA1 and rBcSA1) expressed in *Escherichia coli* were purified and injected into mice for production of anti-sera. The native proteins were characterized by Western blot analysis and immunofluorescence. Furthermore, indirect enzyme-linked immunosorbent assays (iELISA) and rapid immunochromatographic tests (ICT) based on rBcMSA1 or rBcSA1 were established and evaluated to test specific antibodies in consecutive plasma samples from two *B. canis*-infected dogs.

**Results:**

Antiserum raised against rBcMSA1 and rBcSA1 recognized the 39 kDa and 44 kDa native proteins by Western blot analysis, respectively. In addition, immunofluorescence and confocal microscopic observations revealed that BcMSA1 was found on the surface of parasites. However, BcSA1 localized in the matrix of the merozoites. The ELISA and ICT based on rBcMSA1 or rBcSA1 could detect specific antibodies in consecutive plasma samples from two *B. canis*-infected dogs. They showed no cross-reactions against the serum samples collected from dogs experimentally infected with closely related parasites.

**Conclusion:**

Taken together, the current results indicated that the rBcMSA1 and rBcSA1 are promising serodiagnostic antigens for developing iELISA and ICT to detect *B. canis* infection. To our knowledge, this study is the first to report BcMSA1 and BcSA1 as potential antigenic proteins for serodiagnosis of *B. canis* infection in dogs.

## Background

*Babesia canis* is an apicomplexan tick-transmitted protozoan responsible for causing piroplasmosis in dogs [1]. In many regions of Europe and west Asia, this parasite has been reported as the important and frequent causative agent of canine babesiosis [2, 3]. A number of new endemic areas of *B. canis* infection had been reported in European countries, recently [4]. *Babesia canis* is transmitted by *Dermacentor reticulatus* ticks. The symptoms of *B. canis* infection include: anorexia, lethargy, jaundice, fever, anemia and lymphadenopathy [5]. Currently, control of canine babesiosis relies on drug therapy and limited vector control measures. However, drug therapy often relieves symptoms of the infection without clearing the parasites from the dog’s system. Therefore, prompt diagnosis of *B. canis*-infected dogs is important to prevent the disease.

Microscopic examination of Giemsa-stained blood smears is still the reasonably sensitive tool for the diagnosis of canine babesiosis during acute infection. This depends on detection of intraerythrocytic *Babesia* organisms, but it is difficult to detect the parasites during the chronic stage. Serodiagnostic methods are useful for detecting subclinical infection with significantly low level of parasitemia. In general, indirect immunofluorescence assay (IFAT) and indirect enzyme-linked immunosorbent assay (iELISA) are commonly used diagnostic methods to detect chronic *B. canis* infections [6–8]. The above tests are highly sensitive although use of whole parasite antigen derived from infected erythrocytes possibly yields false-positive results with closely related parasites due to cross reaction [9]. By comparison, iELISA and immunochromatographic test (ICT) using recombinant antigens have advantages because they are relatively stable and have higher specificity than parasite-infected erythrocyte antigen-based tests. Although several iELISA and ICT using purified recombinant antigens have been established for a range of protozoan diseases [10, 11], no recombinant antigen-based iELISA and ICT are available for the serodiagnosis of *B. canis*-infected dogs. Therefore, identification and characterization of novel *B. canis* antigens are urgently needed for developing high quality recombinant antigen-based diagnostic tests for detection of *B. canis* infection.

Based on the above background, we constructed a complementary DNA (cDNA) library of *B. canis* and serologically screened the cDNA expression library. A merozoite surface antigen (BcMSA1) and a secreted antigen of *B. canis* (BcSA1) were identified and characterized and their potential as candidates for serodiagnosis was evaluated by both iELISA and ICT.

## Methods

### Parasites and experimental animals

A field strain of *B. canis* was isolated from a clinical case located at the Germany-Austria border and stored under liquid nitrogen. The species was verified by PCR assay for specific discrimination of *B. canis*, *Babesia rossi* and *Babesia vogeli* as described previously [12]. Two one-year-old splenectomized beagle dogs (Nihon Nosan, Japan) were intravenously injected with 1 × 10^7^*B. canis*-infected erythrocytes. Upon attaining 5–8 % parasitemia, blood was collected from the two dogs into tubes containing *potassium* ethylenediaminetetraacetic acid (EDTA) anticoagulant, and stored at -80 °C for total RNA extraction. Another two nonsplenectomized beagle dogs were intravenously infected with 1 × 10^7^*B. canis*-infected erythrocytes. The parasitemia of each dog was examined every day and the blood was collected for preparing the consecutive plasma samples. Giemsa-stained thin blood films were prepared daily to monitor the parasitemia in infected dogs. The blood samples from two nonsplenectomized dogs were serially collected until 80 or 222 days post-infection (dpi) by using vacuum tubes with EDTA anticoagulant. The plasma was separated from RBC by centrifugation at 1,000 *g* for 10 min, both portions were harvested and stored at approximately -70 °C until use.

### Ethical approval

Handing of experimental animals was carried out in accordance with the guide for the care and use of laboratory animals of the National Institutes of Health. The research protocol was approved (Permit Number: 201109–5) by the Animal Care and Use in Research Committee Promulgated by Obihiro University of Agriculture and Veterinary Medicine, Japan. During this study, all animal surgeries were performed under sodium pentobarbital anesthesia and all efforts were made to minimize suffering.

### Construction of cDNA expression library

Total RNA was extracted from 36 ml *B. canis-*infected dog erythrocytes by acid guanidinium thiocyanate phenol chloroform extraction methods as described previously [13]. The polyadenylated mRNA was isolated by Oligotex-dT 30 (JSR and Nippon Roche, Japan) from 1.15 mg of total RNA. The mRNA was precipitated with 3 M sodium acetate and 99.5 % ethanol, after that the mRNA was washed with 70 % ethanol. Next, using 10 ug mRNA, double stranded complementary DNA was synthesized by primers containing Xho I and oligo (dT) sequence from ZAP-cDNA kit, and ligated with Uni-ZAP XR vector. Subsequently the ligation products were packaged by using ZAP-cDNA express GigapackIII Gold cloning kit (Stratagene, USA) in vitro. The cDNA library titer was determined by incubating 10 μl serial dilutions of the recombinant bacteriophage with 200 μl of the XL1-Blue cells (OD_600_ = 0.5) at 37 °C. The titer of cDNA expression library constructed was calculated as follows: pfu/ml = (number of plaques × dilution factor × 10^3^ μl/ml)/(μl of diluted phage plated).

### Serological analysis of cDNA expression library

The *B. canis*-infected dog sera were used to immune-screen the cDNA expression library. The cDNA library was plated at a density of 1.5 × 10^4^ plaque-forming units (PFUs) per 100 cm^2^ square plate on a total of 20 plates. The phage plaques were overlaid with nitrocellulose membranes and incubated with the *B. canis*-infected dog serum following instruction of the PicoBlue Immunoscreening Kit (Stratagene, USA). Briefly, the nitrocellulose membranes were blocked with 1 % bovine serum albumins (BSA) diluted in Tris-buffered saline (TBS) and then incubated in a 1:100 dilution of anti-*B. canis* antibodies for 2 h. After washing 5 times with TBS containing 0.05 % Tween20 (TBST) for 5 min, the membranes were treated with 1:2000 diluted alkaline phosphatase conjugate goat anti-dog IgG (Bethyl Laboratories Inc., Montgomery, TX, USA) for 1 h. Then the membranes were washed 4 times with TBST and once with TBS. For color development, the membranes were soaked in the fresh substrate (0.3 % nitroblue tetrazolium (NBT) and 5-bromo-4-chloro-3-indolyl phosphate (BCIP) (Roche, Switzerland) and kept in the dark for 2 min. The membranes were then rinsed with TBS, dried at room temperature and the positive plaques were identified on the plate. In order to isolate the single plaque, all the positive plaques were rescreened three times, and the positive plaques were selected. Finally, the cDNA inserts in the lamda vectors were converted into phagemid vectors after in vivo excision.

### DNA sequencing of BcMSA1 and BcSA1 cDNA

The plasmid inserts were rescued in pBluescript SK (pBSK) by in vivo excision technique using ExAssist Interference-Resistant Helper Phage with the *E. coli* SOLR strain following the manufacturer’s instructions (Stratagene, USA). The recombinant plasmids were sequenced using M13 forward, reverse and internal primers, respectively. The sequence was analyzed and assembled by using Genetyx software version 7.0. The sequence data of the BcMSA1 and BcSA1 was submitted to the GenBank with the accession numbers KR134351 and KR134352, respectively. The amino acid sequences of BcMSA1 and BcSA1 were analyzed by a glycosylphosphatidylinositol (GPI) anchor predictor (FragAnchor, http://navet.ics.hawaii.edu/∼fraganchor/NNHMM/NNHMM.html). The secondary structure of BcMSA1 amino acid sequence was predicted by the SOSUI system (http://harrier.nagahama-i-bio.ac.jp/sosui/).

### Southern blot analysis

Genomic DNA of *B. canis* was extracted from parasite-infected erythrocytes for Southern blotting. Five micrograms of DNA was used for each restriction digestion analysis using enzymes that did not cut the probe-specific sequence of BcMSA1 (*Bam*HI, *Sal*I) and BcSA1 (*Bam*HI, *Sac*I). Furthermore, the DNA was also digested with the restriction enzymes that cut a single site within the probe-specific sequence of BcMSA1 (*Pac*I, *Kpn*I and *Nae*I) and BcSA1 (*Nhe*I and *Kpn*I), respectively. The digested DNA samples were submitted to 1 % (w/v) agarose gel electrophoresis. Then, the fractionated DNA was transferred to Hybond-N+ nylon membrane (Amersham-Buchler, Germany). The BcMSA1 and BcSA1 probes that were PCR-amplified using specific primers were directly labeled with alkaline phosphatase (GE Healthcare Bio-Science Co.) and hybridized following the manufacturer’s instructions. The filters were pre-hybridized at 56 °C for 6 h, and hybridization was carried out at 56 °C for 12 h in a hybridization oven with labeled probes of full-length of BcMSA1 and BcSA1, respectively. The blots were washed for several times. Chemiluminescence signals were generated using the CDP-Star detection reagent (GE Healthcare Bio-Science Co.).

### Cloning, expression and purification of rBcMSA1 and rBcSA1 in *E. coli*

The cDNA fragment of BcMSA1 lacking signal peptide and truncated gene of BcSA1 was amplified, respectively, using two pairs of primers: 5′-CGGGATCCGAAAACACTATACTTTTATCC-3′ and 5′-CGGTCGAC TTATTAAAGTTTAGGAGAAGCAGCAGT-3 for BcMSA1 gene; 5′-CGGAATTCCAATCAACAAGCAGCCAG-3′, and 5′-CGGTCGACCTAGTTGATTCATTCTTA-3′ for BcSA1 gene. After restriction enzyme digestion, the PCR products were ligated into the pGEX-4 T vector that was digested by the same restriction enzymes. After ligation, the plasmid was transformed into *E. coli*. The transformed colonies were cultured in LB broth medium with ampicillin sodium (100 μg/ml) at 37 °C, when the OD_600_ value reached 0.5, isopropylthio-beta-D-galactosid (IPTG) was added into logarithmically growing bacterial culture to induce expression of the recombinant BcMSA1 and BcSA1 for 6 h. After centrifugation, the bacteria were harvested and re-suspended in the lysis buffer (100 mM sodium phosphate, pH 8.0; 10 mM Tris-Cl, pH 8.0), and then sonicated for 10 min. Glutathione S-transferase (GST)-tagged recombinant proteins were incubated with glutathione-Sepharose 4B beads (Amersham Pharmacia Biotech, USA) at 4 °C for overnight. The recombinant proteins were recovered and the GST tag was cleaved and removed by thrombin protease. Finally, the concentrations of purified protein samples were detected by the modified Lowry protein assay kit (Thermo Scientific, USA).

### Production of anti-BcMSA1 and anti-BcSA1 sera

Five 6- to 7-week-old female ICR mice (Clea, Tokyo, Japan) were immunized intraperitoneally (i.p.) with 100 μg of purified rBcMSA1-GST or rBcSA1-GST in an equal volume of Freund’s complete adjuvant (Difco Laboratories, USA) for the primary immunization, respectively. Two booster immunizations were given at 14 day intervals by i.p. using 100 μg of the same protein emulsified in Freund’s incomplete adjuvant. Ten days after the last booster shot, the whole blood was collected and serum was harvested and stored at -30 °C.

### Sodium dodecyl sulphate polyacrylamide gel electrophoresis (SDS-PAGE) and Western blot analysis

The recombinant BcMSA1 and BcSA1 proteins were verified by 10 % SDS-PAGE. Furthermore, mouse anti-rBcMSA1 and anti-rBcSA1 sera were used to detect the native BcMSA1 and BcSA1 from *B. canis*-infected erythrocytes and plasma by Western blot analysis. The *B. canis*-infected erythrocytes and plasma (10-time concentrated) obtained from infected splenectomized dogs with 8 % parasitemia were sonicated in a loading buffer and were heated at 100 °C for 10 min. Then the proteins were size-separated by electrophoresis in 10 % SDS-PAGE. Afterwards, the proteins were transferred onto a nitrocellulose membrane for 1 h at 60 mA and blocked in 5 % PBS skim-milk for 2 h at room temperature. The blots were incubated with anti-BcMSA1 and anti-BcSA1 polyclonal antibody (1:100) for 1 h. After washing 5 times, the membranes were incubated with horseradish peroxidase conjugated anti-mouse IgG (1:2,500) for 1 h at room temperature. The peroxide activity was visualized with 0.05 % (w/v) 3,3′-diaminobenzidine tetrahydrochloride in 50 Mm Tris–HCl buffer (pH 7.2) containing hydrogen peroxide for 2 min. To determine the antibody responses against BcMSA1 and BcSA1 in *B. canis*-infected dogs, the rBcMSA1 and rBcSA1 proteins were subjected to SDS-PAGE analysis. After electrophoresis, the proteins were transferred onto the membrane and probed with 100 times diluted *B. canis*-infected dog sera and pre-infected dog sera, respectively. The blots were used for western blotting as described above.

### Immunofluorescent antibody test (IFAT) and confocal laser microscopy observations

Thin blood smears were prepared from *B. canis*-infected red blood cells. The smears were air-dried, and fixed in 30 % acetone-70 % methanol solution for 30 min at -30 °C. The fixed smears were incubated with anti-rBcMSA1 and anti-rBcSA1 sera raised in mice (1:200 dilutions) in 5 % skim milk-PBS for 1 h at 37 °C, respectively. After washing three times with 1 % PBS-Tween 20 (PBST), the smears were incubated with Alexa-Fluor 488-conjugated goat anti-mouse immunoglobulin G (IgG) (Molecular Probes, USA) for another 1 h at 37 °C. The slides were washed three times with PBST, rinsed with PBS and the parasite nucleus was stained with a solution containing propidium iodide (PI) (6.25 mg/ml, Wako, Japan) and RNase A (50 mg/ml, Qiagen, Germany) for 20 min at 37 °C. Finally, the slides were mounted in a Dako cytomation fluorescent mounting medium (Dako, Carpenteria, CA., USA) and examined under a confocal laser scanning microscope (TCSNT, Leica, Germany).

### Indirect enzyme-linked immunosorbent assay (iELISA) based on rBcMSA1 and rBcSA1

Purified GST-rBcMSA1, GST-rBcSA1 and GST (control) were diluted in a coating buffer (0.05 M carbonate-bicarbonate buffer, pH 9.6) to a final concentration of 2 μg/ml. The microtiter plates (Thermo Scientific) were coated with each protein overnight at 4 °C and blocked with 3 % (w/v) skim milk solution for 1 h at 37 °C. After washing, the plates were incubated with 200 times diluted serum samples from dogs experimentally infected with *Neospora caninum*, *B. gibsoni*, *B. rossi*, *B. vogeli*, *B. canis*, 30 canine serum samples from non-endemic area and sequential serum samples from the infected dogs, respectively. The plates were washed 3 times with PBST and incubated with horseradish peroxidase (HRP) conjugated anti-dog IgG (1:4000) for 1 h at 37 °C. After washing, the enzyme reaction was developed with 2, 2-azinobis (3-ethylbenzthiazolinesulfonic acid) (ABTS) (Sigma, USA). The optical density (OD) was measured at wavelength of 415 nm and the cut-off values were defined as the mean value plus 5 × standard deviations of the mean OD value.

### Immunochromatographic test (ICT) based on rBcMSA1 and rBcSA1

The test lines for ICT were prepared by diluting the GST free rBcMSA1 and rBcSA1 separately in PBS to optimal concentration of 200 μg/ml. However, the control line was prepared from purified mice anti-rBcMSA1 or anti-rBcSA1 polyclonal IgG, dissolved in PBS to a concentration of 1,500 μg/ml. Aliquots of the recombinant protein solutions were applied to a nitrocellulose membrane using a BioDot Biojet 3050 quanti-dispenser (BioDot, USA). Colloidal gold-conjugated recombinant proteins containing 7.5 % BSA were applied to a fiberglass membrane and then the membranes were dried at room temperature. The complete test structures were assembled and the multimembrane composite was cut into 2 mm wide strips using a BioDot cutter (BioDot, USA). Detection was performed by pipetting 50 μl of the diluted serum (1:10 in PBS) on the sample pad. The result was judged 30 min after the application of serum samples.

### Serum samples

Canine sera used for ELISA and ICT were as follows: 12 sera from dogs experimentally infected with *B. canis,* 3 sera from dogs experimentally infected with *B. vogeli*, 3 sera from dogs experimentally infected with *B. rossi*, 3 sera from dogs experimentally infected with *B. gibsoni*, 3 sera from dogs experimentally infected with *Neospora caninum*, 30 sera from specific-pathogen-free (SPF) dogs and 30 non-endemic control canine serum samples were collected from non-endemic area in Osaka, Japan, serial serum samples (from 0 to 222 days post-infection) from two dogs experimentally infected with *B. canis*.

## Results

### Isolation and identification of the cDNA encoding BcMSA1 and BcSA1

**Fig. 1:**
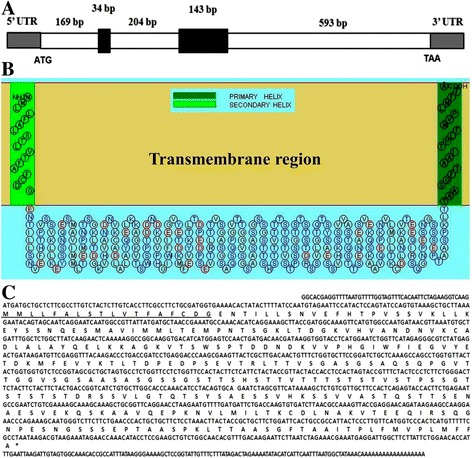
The hypothetical transmembrane helix of BcMSA1 in the membrane using the SOSUI bioinformatic tool. **a** Structure of the genomic BcMSA1 gene. White bold lines indicate exons, and black rectangles indicate introns. The numeric numbers indicate the number of nucleotides; **b** BcMSA1 was predicted to have two hypothetical transmembrane helixes located at the N and C terminal; **c** Nucleotide sequence and predicted amino acid sequences of the genomic BcSA1 gene

Approximately 20,000 plaques from the *B. canis* cDNA expression library were screened with the *B. canis*-infected dog serum. A total of 41 immunoreactive recombinant phage plaques were identified after immunoscreening. These homogeneous plaques expressing the antigens were selected and excised to the pBluescript SK phagemid. Ten of 41 immunoreactive recombinant phage plaques shared the same sequence, which showed no match with other genes by Blast. The full length of the BcMSA1 cDNA sequence has a single open reading frame of 966 nucleotides encoding a polypeptide of 321 amino acids. The theoretical isoelectric point and molecular mass for the mature protein are 4.93 and 33.599 kDa, respectively. Genomic analyses indicated that BcMSA1 gene contains two introns and three extrons (Fig. 1a). The predicted transmembrane region of BcMSA1 was determined according to the computed algorithm (Fig. 1b). Kyte-Doolittle’s hydropathy analysis indicated that the amino acid sequence of BcMSA1 has a hydrophilic core region with a good antigenic index, which suggested that the antigen could be a good candidate for detection of *B. canis* antibodies (data not shown). GPI anchor prediction indicated that three of highly probably GPI sequences were involved in BcMSA1. One GPI sequence located from 297–321 of this amino acid sequence, and another two located from 304–321 and 305–321, respectively. Furthermore, another novel gene named BcSA1 was also identified from these 41 immunoreactive recombinant phage plaques. The partial length of BcSA1 contained 1,255 nucleotides encoding a polypeptide with 417 amino acid residues (Fig. 1c). The isoelectric point and molecular mass for the mature protein without signal peptide were 8.77 and 44.06 kDa, respectively. Unlike BcMSA1, the BcSA1 had not predicted GPI-anchored domains in its amino acid sequence.

### Southern blot analysis

**Fig. 2:**
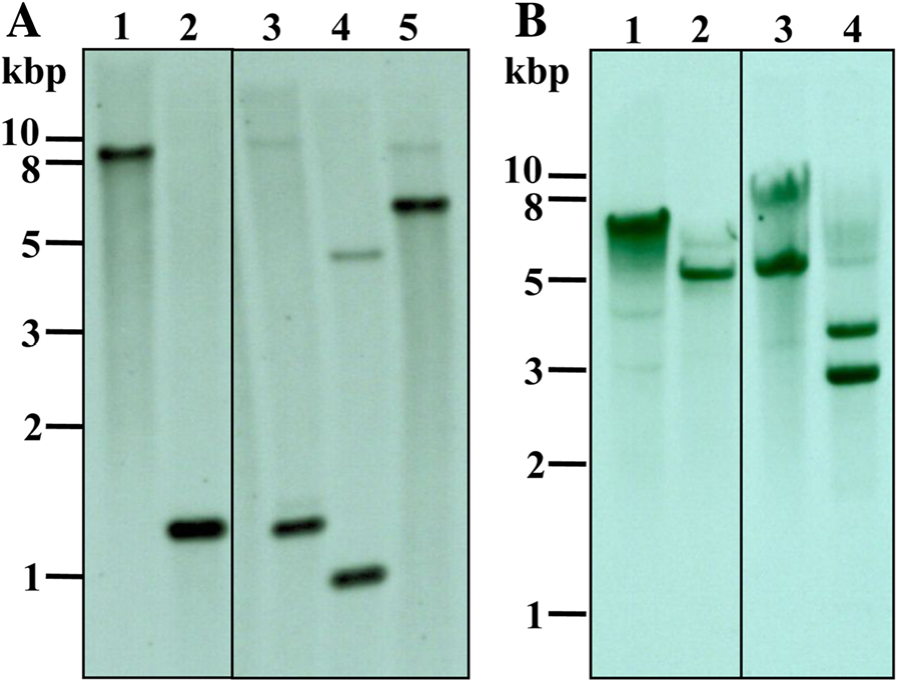
Southern blot analysis of genomic DNA of *Babesia canis*. **a** Genomic DNA was digested with different restriction enzymes, *Bam*HI (lane 1), *Sal*I (lane 2), *Pac*I (lane 3), and *Kpn*I (lane 4) and *Nae*I (lane 5), and hybridized with specific probe of the BcMSA1 gene; **b** Genomic DNA was digested with different restriction enzymes, *Bam*HI (lane 1), *Sac*I (lane 2), *Nhe*I (lane 3), and *Kpn*I (lane 4), and hybridized with a specific probe of the BcSA1 gene

Southern blot analysis was performed to determine the copy number of BcMSA1 and BcSA1 gene, respectively. A probe derived from cDNA clone BcMSA1 was strongly hybridized to the *B. canis* DNA fragments, as shown in Fig. 2a. Treatment with the restriction enzymes (*Bam*HI and *Sal*I) that do not cut within the sequence consistent with the probe produced a single band (Fig. 2a, lanes 1, 2). However, two bands were observed after treatment with enzymes (*Pac*I, *Kpn*I and *Nae*I) that cut a single position with the probe sequence (Fig. 2a, lanes 3–5). These results revealed that the genomic DNA of *B. canis* contains a single-copy gene encoding BcMSA1. Similar result was also observed for BcSA1 cDNA with the restriction enzymes that cut once within the gene (Fig. 2b, lanes 3, 4) and those that did not cut with the gene (Fig. 2b, lanes 1, 2). These results also indicate that the BcSA1 gene exists as a single copy in *B. canis* genome.

### Expression, purification and Western blot analysis of rBcMSA1 and rBcSA1

**Fig. 3:**
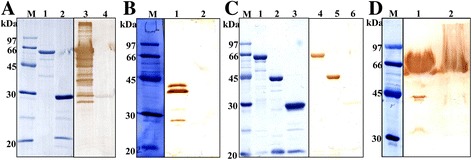
SDS-PAGE and immunoblot analysis of recombinant and native BcMSA1 and BcSA1. **a** The 10 % SDS-PAGE stained with Coomassie blue: recombinant BcMSA1 fused with GST (lane 1) and GST (lane 2). Western blot analysis of recombinant protein: recombinant BcMSA1 (lane 3) and GST (lane 4) probed with *B. canis*-infected dog serum; **b** Western blot analysis of native BcMSA1. *B. canis*-infected erythrocyte lysate (lane 1) and normal canine erythrocyte lysate (lane 2) probed with anti-rBcMSA1 mouse serum; **c** The 10 % SDS-PAGE stained with Coomassie blue: recombinant BcSA1 fused with GST (lane 1), recombinant BcSA1 fused without GST (lane 2) and GST (lane 3). Western blot analysis of recombinant protein: recombinant BcMSA1 (lane 4), recombinant BcSA1 fused without GST (lane 5) and GST (lane 6) probed with *B. canis*-infected dog serum; **d** Western blot analysis of native BcMSA1. The plasma from a dog infected with *B. canis* (lane 1) and a normal dog (lane 2) probed with anti-rBcMSA1 mouse serum

The amplified BcMSA1 and BcSA1 genes were cloned into the prokaryotic expression vector pGEX-4 T-1, respectively. Both BcMSA1 and BcSA1 were expressed in *E. coli* as soluble GST-fusion proteins with molecular mass of approximately 64 kDa (Fig. 3a, lane 1; Fig. 3c, lane 1). Western blot analysis showed that serum antibodies from dogs experimentally infected with *B. canis* could react with both of the recombinant fusion proteins (Fig. 3a, lane 3; Fig. 3c, lanes 4, 5). In contrast, no reaction of the serum was obtained with the GST protein (Fig. 3a, lane 4; Fig. 3c, lane 6).

### Characterization of the native BcMSA1 and BcSA1 of *B. canis*

**Fig. 4:**
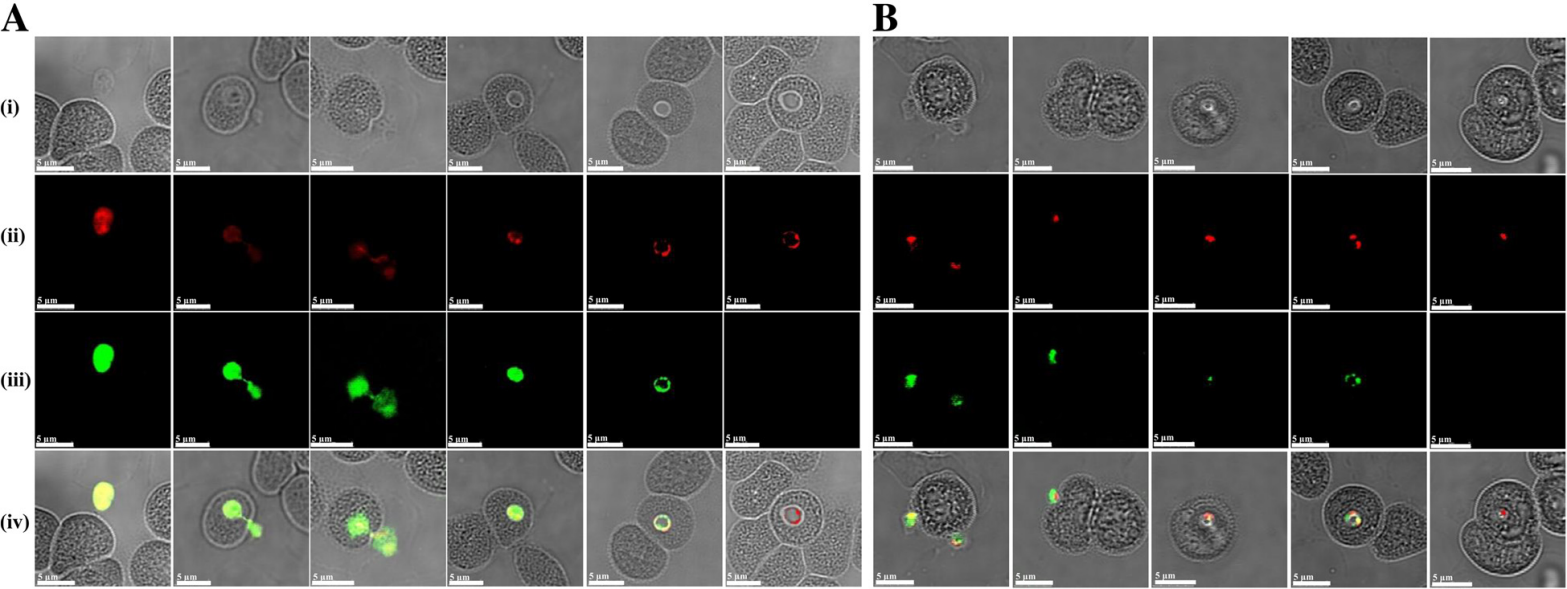
Localization of BcMSA1 and BcSA1 at inner and outer surface of erythrocyte stages of *B. canis* by immunofluorescence staining and laser confocal microscopy. **a** Observation of the native BcMSA1 in thin blood smears of *B. canis*-infected RBC stained with mice anti-rBcMSA1 serum and pre-immune serum; **b** Observation of the native BcSA1 in thin blood smears of *B. canis*-infected RBC stained with mice anti-rBcSA1 serum and pre-immune serum: (i) Phase-contrast images; (ii) Propidium iodide staining; (iii) Immunofluorescent staining; (iv) Merged image

Mouse anti-rBcMSA1 and anti-rBcSA1 polyclonal sera were prepared and used to identify native BcMSA1 and BcSA1 in *B. canis* parasites by Western blot analysis and confocal laser scanning microscopy. As shown in Fig. 3b the 39 kDa band was detected with mouse anti-rBcMSA1 sera in *B. canis*-infected erythrocyte lysate (Fig. 3b, lane 1). There was no reaction for the normal erythrocyte lysate with the anti-rBcMSA1 serum (Fig. 3b, lane 2). On the other hand, the predicted 44 kDa band was detected by mouse anti-rBcSA1 sera in the plasma from *B. canis*-infected dog during acute stage of infection (Fig. 3d, lane 1). Furthermore, the mouse anti-rBcMSA1 serum did not react with the plasma of *B. canis*-infected dog and the mouse anti-rBcSA1 serum did not react with *B. canis*-infected erythrocyte lysate in Western blot analysis (data not shown). Native protein localization studies by IFAT revealed that BcMSA1 and BcSA1 possibly localizes on the cell surface (Fig. 4a) and within the cytoplasm of the parasite (Fig. 4b), respectively.

### Evaluation of the serodiagnostic potential of rBcMSA1 and rBcSA1 by iELISA

**Fig. 5:**
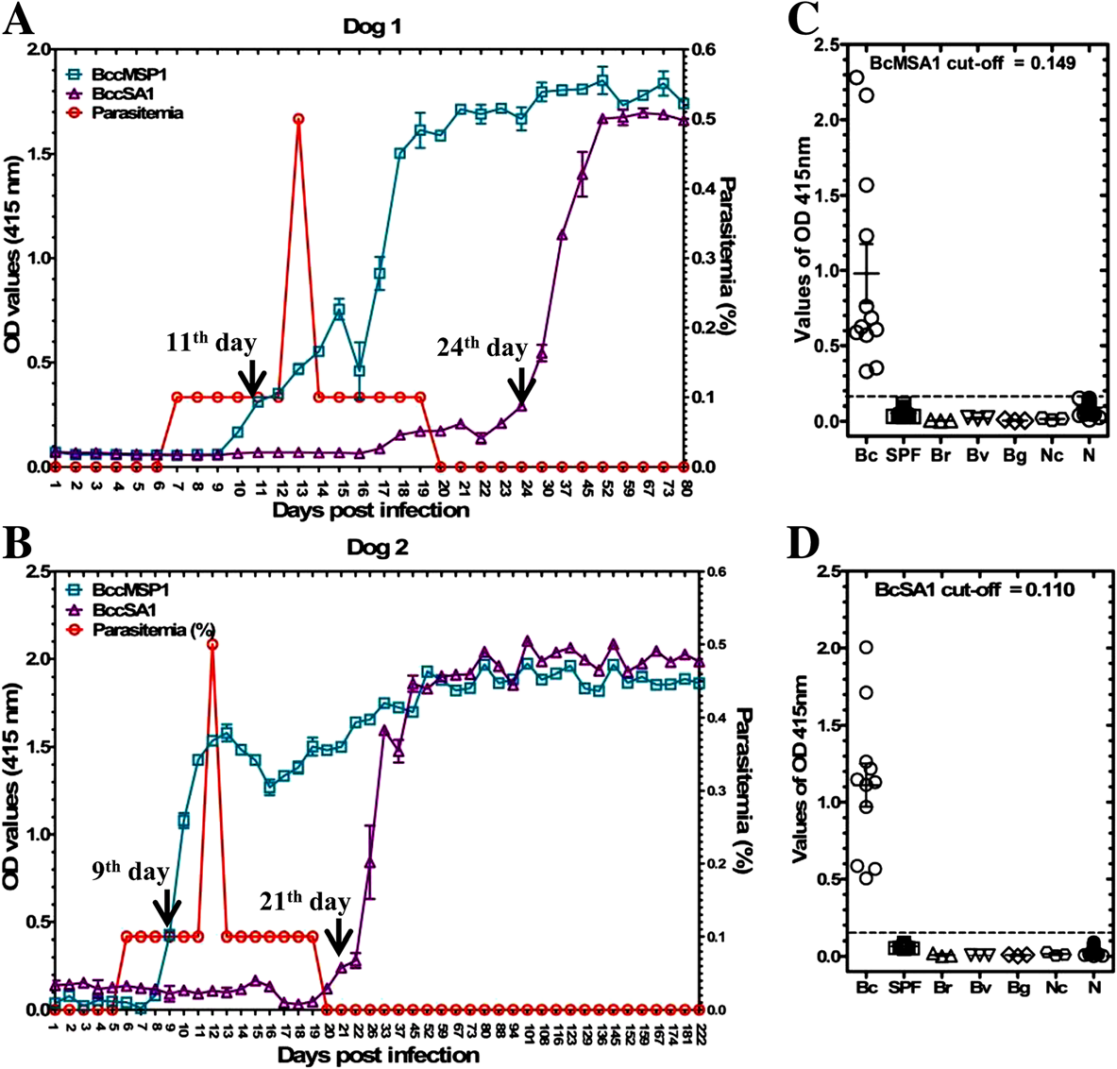
Evaluation of rBcMSA1 and rBcSA1 as diagnostic antigens by ELISA system. **a**, **b** Antibody responses of sequential serum samples from two non-splenectomized dogs experimentally infected with *B. canis* to rBcMSA1 and rBcSA1 antigens in iELISA; **c**, **d** The reactivity of rBcMSA1 and rBcSA1 with *B. canis* and closely related parasite-infected dog sera. *Abbreviations*: Bc, *B. canis-*infected dogs sera; SPF, SPF dog sera; Br, *B. rossi*-infected dogs sera; Bv, *B. vogeli*-infected dogs sera; Bg, *B. gibsoni-*infected dogs sera; Nc, *N. caninum*-infected dogs sera; N, serum samples from non-endemic area in Osaka, Japan

The rBcMSA1- and rBcSA1-based ELISA experiments were conducted to investigate whether these recombinant proteins could be used for diagnosis of *B. canis* infection in dogs. The cut-off values of BcMSA1-ELISA and BcSA1-ELISA were calculated using 30 SPF canine sera; these were 0.149 and 0.110, respectively (Fig. 5c, d). As shown in Fig. 5c and d, all the serum samples from dogs infected with *B. canis* were positive for these two antigens, whereas, all 30 canine serum samples from non-endemic area (*N*) were negative. The serum samples from *B. rossi*-infected dogs (*Br*), *B. vogeli*-infected dogs (*Bv*), *B. gibsoni-*infected dogs (*Bg*) and *N. caninum*-infected dogs (*Nc*) were negative for the antibodies against BcMSA1 and BcSA1. The sensitivity of these assays were tested with sequential sera obtained from two dogs experimentally infected with *B. canis*, both serologically negative prior to infection. For the BcMSA1-ELISA, the IgG titers increased in one dog on day 9 post-infection and in another dog on day 11 post-infection. Both dogs were serologically positive until 80 days post-infection (Fig. 5a, b). Towards day 80, the dogs already entered the chronic phase of the disease, evidenced by recovering hematocrit value (data not shown) and significantly low levels of parasitemia. In contrast, specific antibodies against BcSA1 were detected on day 24 post-infection in one dog and on day 21 post-infection in another dog. Interestingly, the two dogs were serologically positive for BcSA1 throughout the sampling period.

### Evaluation of the serodiagnostic potential of rBcMSA1 and rBcSA1 by ICT

**Fig. 6:**
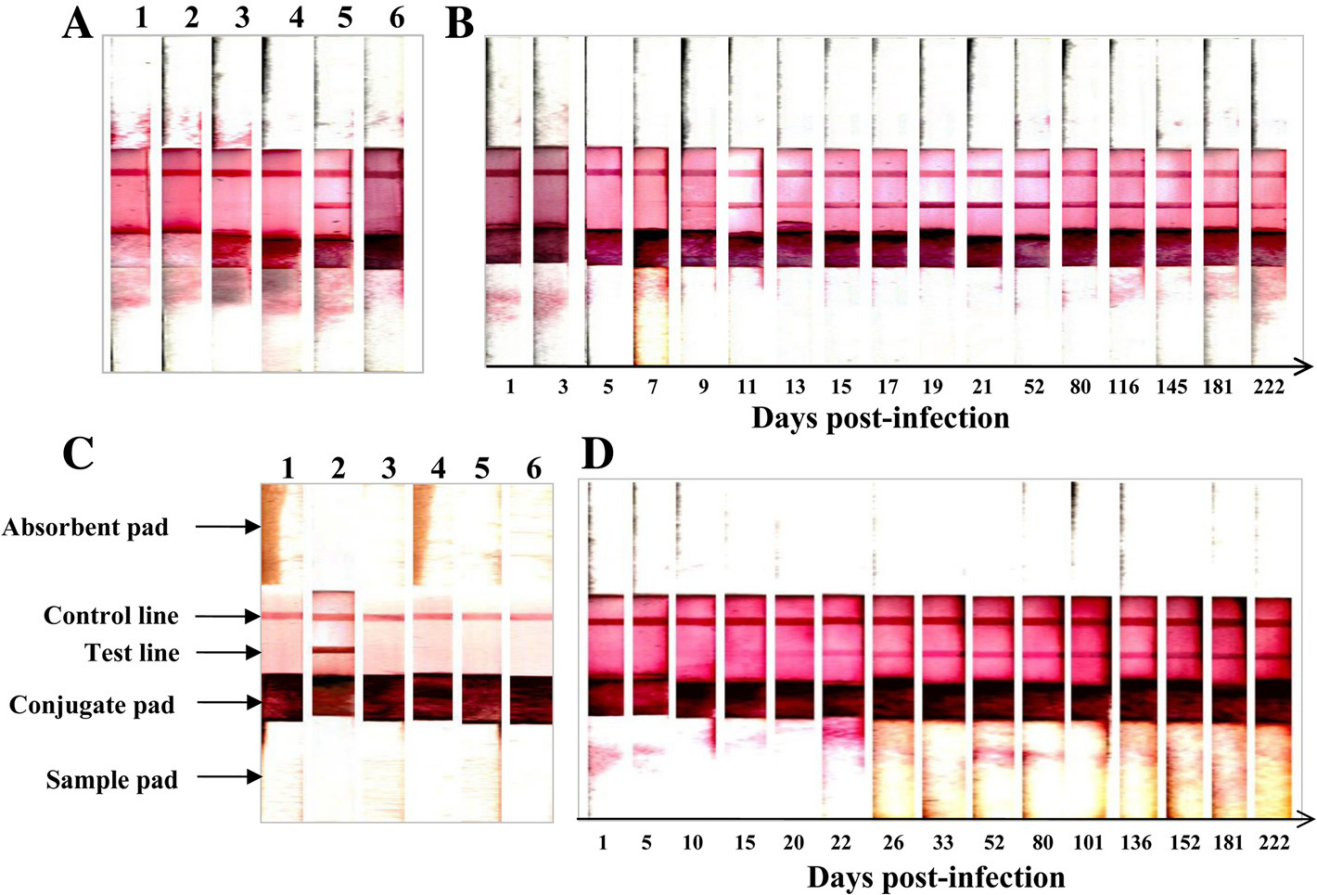
Evaluation of ICT based on rBcMSA1 and rBcSA1. **a** Cross-reactivity of ICT using rBcMSA1 with closely related parasite-infected canine sera: lane 1, *B. rossi*-infected dog serum; lane 2, *B. vogeli*-infected dog serum; lane 3, *B. gibsoni*-infected dog serum; lane 4, *L. infantum*-infected dog serum; lane 5, *B. canis-*infected dog serum; lane 6, a SPF dog serum; **b** Specific antibody responses to rBcMSA1 in sequential serum samples from a non-splenectomized dog experimentally infected with *B. canis*; **c** Cross-reactivity of rBcSA1 with closely related parasite-infected canine sera: lane 1, SPF dog serum; lane 2, *B. canis*-infected dog serum; lane 3, *B. rossi*-infected dog serum; lane 4, *B. vogeli*-infected dog serum; lane 5, *B. gibsoni*-infected dog serum; lane 6, *L. infantum*-infected dog serum; **d** Specific antibody responses to rBcSA1 in sequential serum samples from a non-splenectomized dog experimentally infected with *B. canis*

The performance of rBcMSA1 and rBcSA1 as rapid and simple diagnostic antigens for detection of *B. canis* infection in dogs was evaluated using ICT. Serum samples from experimentally infected dogs with closely related parasites (*B. rossi*, *B. vogeli*, *B. gibsoni* and *N. caninum*) were used to determine the specificity of ICT based on rBcMSA1 and rBcSA1. Only the serum samples from *B. canis*-infected dogs were positive in the ICT, the other serum samples and SPF dog sera were negative (Fig. 6). As shown in Fig. 6, the specific antibodies to BcMSA1 and BcSA1 could be detected from day 9 and day 21, respectively. The antibodies against BcMSA1 and BcSA1 were detectable until 80 days post-infection, which was consistent with the results of the iELISA.

## Discussion

Canine babesiosis frequently caused by *Babesia canis* is an emerging infectious disease in central Europe [14, 15]. A number of new endemic regions of this disease have been reported in European countries and beyond [16–19]. Canine babesiosis also represents an important veterinary medical problem, since to date treatment and diagnosis of this disease is still limited and the current vaccine is derived from culture supernatants [20–22]. Therefore, it is critical to have sensitive and reliable tests for screening the *B. canis* positive population for prompt intervention. In this regard, we attempted to identify novel antigens to develop reliable, rapid and sensitive diagnostic methods for *B. canis* infections. A cDNA expression library was constructed from *B. canis* merozoites mRNA and serologically screened with *B. canis-*infected serum, and novel BcMSA1 and BcSA1 were isolated and identified in this study.

Homology blast result indicated that BcMSA1 shared no significant homology with any of the apicomplexan parasites, which was important to confer a degree of specificity when used as a diagnostic probe. A 39 kDa native protein of *B. canis* merozoites was recognized in Western blot analysis. The molecular mass of this native protein was higher than predicted. The difference in the molecular mass could be attributed to glycosylation modification since two predicted N-glycosylation sites were identified in BcMSA1 motif by the NetNGlyc predictor or the PROSITE pattern PS00001 (data not shown). Furthermore, BcMSA1 was identified as a surface protein of *B. canis* merozoites. Generally, the merozoite surface proteins of *Babesia* spp. are thought to be the main targets for the host immune responses. For the *Babesia* spp., merozoite surface proteins are usually involved in the merozoite invasion of host erythrocytes and provide potential targets for vaccine and serodiagnosis [23–26]. Therefore, BcMSA1 might be a potential candidate for diagnostic antigen and vaccines for diagnosis and prevention of *B. canis* infection, respectively. However, the merozoite surface antigens of *Babesia* spp. are genetically diverse among the different isolates [24, 25]. Thus, more sequences of BcMSA1 from different regions should be identified for further study.

Sequence analysis of BcSA1 revealed that this antigen contained a partial of signal peptide sequence, and BcSA1 shared 25 % amino acid identity with *B. gibsoni* secreted antigen 1 (BgSA1) in previous report [27]. Furthermore, a 44 kDa native protein was detected by Western blot, and this was consistent with the molecular mass of BcSA1 without signal peptide. Therefore, the results indicated that BcSA1 may represent a secreted protein of *B. canis*. Secreted proteins have been proved as suitable sources of antigens for the detection of parasites infection in previous studies [27–31]. Therefore, identification of BcSA1 will be very useful in the development of effective diagnostic methods for the detection of circulating antigens and antibodies during *B. canis* infection. In addition, another unexpected 69 kDa band was observed in the 10-time concentrated *B. canis*-infected dog plasma from both infected and non-infected dogs in the Western blot analysis, which might be dog IgG cross-reacted with the second antibody to give an unspecific band.

To determine the diagnostic potential of the rBcMSA1 and rBcSA1 in the serological assays, the recombinant proteins were used as antigens in iELISAs. The results of specificity analysis indicated that both BcMSA1 and BcSA1 were specific for *B. canis* infection. This makes the two antigens become suitable candidates for diagnosis of *B. canis* infection by iELISA. On the other hand, BcMSA1-ELISA and BcSA1-ELISA could detect the specific antibody as early as 9 and 22 days post-infection, respectively. Moreover, the antigen-antibody interactions were still detectable until the chronic stage of infection with both antigens. These results indicated that both rBcMSA1 and rBcSA1 can detect chronic *B. canis* infection in dogs and the BcMSA1 can detect *B. canis* during early infection. The ability of BcMSA1 and BcSA1 to induce strong humoral immunity in *B. canis*-infected dogs necessitates further evaluation of the recombinant antigens as vaccine candidates against *B. canis* infection.

Immunochromatographic test (ICT) is a one-step rapid diagnostic method, which is suitable for various clinical environment and can be completed in a short time (10–15 min) [32, 33]. Therefore, in this study, we established ICT methods for detection of *B. canis* infection. The ICT based on rBcMSA1 or rBcSA1 could detect the specific antibodies from dogs with *B. canis *infection. These results suggest that rBcMSA1 and rBcSA1 could be candidate antigens for the development of ICT to detect *B. canis* infection.

## Conclusion

In this study, a *B. canis* cDNA expression library was constructed and immunoscreened using *B. canis*-infected dog sera. This study was the first to identify and characterize two novel antigens of *B. canis* encoding a merozoite surface antigen (BcMSA1) and a secreted protein (BcSA1) as potential serodiagnostic antigens. Our results demonstrated that both, rBcMSA1 and rBcSA1, were highly sensitive and specific for serodiagnosis of *B. canis* infection. Taken together, rBcMSA1 and rBcSA1 could be the first promising serodiagnostic antigens based on iELISA and ICT for detecting *B. canis* infection in dogs.
